# Blocking downstream signaling pathways in the context of HDAC inhibition promotes apoptosis preferentially in cells harboring mutant Ras

**DOI:** 10.18632/oncotarget.12001

**Published:** 2016-09-13

**Authors:** Julian C. Bahr, Robert W. Robey, Victoria Luchenko, Agnes Basseville, Arup R. Chakraborty, Hanna Kozlowski, Gary T. Pauly, Paresma Patel, Joel P. Schneider, Michael M. Gottesman, Susan E. Bates

**Affiliations:** ^1^ Developmental Therapeutics Branch, National Cancer Institute, National Institutes of Health, Bethesda, MD 20892, USA; ^2^ Laboratory of Cell Biology, National Cancer Institute, National Institutes of Health, Bethesda, MD 20892, USA; ^3^ Chemical Biology Laboratory, National Cancer Institute, Frederick Cancer Research Center, Frederick, MD 21702, USA; ^4^ Columbia University Medical Center, Division of Hematology/Oncology, New York, NY 10032, USA

**Keywords:** romidepsin, Ras mutation, apoptosis, MEK inhibitor, AKT inhibitor

## Abstract

We previously demonstrated activation of the mitogen-activated protein kinase (MAPK) pathway in a series of romidepsin-selected T-cell lymphoma cell lines as a mechanism of resistance to the histone deacetylase inhibitor (HDI), romidepsin. As Ras mutation leads to activation of both the MAPK and the phosphoinositide 3-kinase (PI3K) pathway, we examined whether combining romidepsin with small molecule pathway inhibitors would lead to increased apoptosis in cancers harboring Ras mutations. We treated 18 Ras mutant or wild-type cell lines with romidepsin in the presence of a MEK inhibitor (PD-0325901) and/or an AKT inhibitor (MK-2206) and examined apoptosis by flow cytometry. A short-term treatment schedule of romidepsin (25 ng/ml for 6 h) was used to more closely model clinical administration. Romidepsin in combination with a MEK and an AKT inhibitor induced apoptosis preferentially in cells harboring mutant versus wild-type Ras (69.1% vs. 21.1%, *p* < 0.0001). Similar results were found in a subset of cell lines when belinostat was combined with the MEK and AKT inhibitors and when romidepsin was combined with the dual extracellular signaling-related kinase (ERK)/PI3K inhibitor, D-87503, which inhibited both the MAPK and PI3K pathways at 5–10 μM. The observed apoptosis was caspase-dependent and required Bak and Bax expression. Cells with wild-type or mutant Ras treated with romidepsin alone or in combination with the MEK inhibitor displayed increased expression of proapoptotic Bim. We thus conclude that cancers bearing Ras mutations, such as pancreatic cancer, can be targeted by the combination of an HDI and a dual inhibitor of the MAPK and PI3K pathways.

## INTRODUCTION

Mutations in the Ras isoforms HRAS, KRAS, or NRAS occur in approximately 30% of human cancers, with KRAS mutations being the most prevalent occurring in over 90% of pancreatic cancers and 32% of lung cancers [1–4]. Activating mutations in Ras lead to a perpetual catalytically “on” state, allowing unrestricted activation of downstream effector pathways including Rac, Ral, Raf, protein kinase C (PKC) and phosphoinositide 3-kinase (PI3K). It is by activation of these pathways that mutant Ras drives both tumor growth and imparts drug resistance, leading to an intense search for strategies to target Ras.

Although as yet there are no therapies that specifically target Ras, inhibitors of downstream pathways such as the mitogen-activated protein kinase (MAPK) and PI3K pathways are currently in clinical trials. Activation of these pathways is associated with increased resistance to apoptosis. Extracellular signaling-related kinase (ERK) has been shown to directly phosphorylate and deactivate the proapoptotic protein Bim, targeting the protein for degradation by the proteasome [5–7]. Akt has also been shown to phosphorylate and impair Bim [8] and can additionally phosphorylate Bax, regulating its role in the execution of apoptosis [9, 10]. Despite the fact that several MAPK and PI3K inhibitors have been developed clinically both as single agents and in combination with other therapeutics, significant progress has not been made in the treatment of Ras-mutant cancers.

As our previous work suggested that activation of the MAPK pathway is a mechanism of resistance to the histone deacetylase inhibitor (HDI) romidepsin [11], we examined the efficacy of romidepsin in combination with MAPK inhibition in Ras mutant cancers that harbor constitutive activation of the MAPK pathway, as well as Ras wild-type cancers. Romidepsin has previously been shown to inhibit growth of Ras-transformed cells and, interestingly, it was discovered in a screen targeting Ras [12, 13]. Given that Ras activates the PI3K pathway as well as the MAPK pathway, we also combined romidepsin with an inhibitor of the PI3K pathway. We found striking activity of romidepsin in combination with inhibitors of these downstream effector pathways that was not observed in cell lines with wild-type Ras. However, clinical trials that combine inhibitors of the MAPK and PI3K pathways have led to excessive toxicity [14, 15]. This led us to examine the feasibility of using a dual inhibitor of the two pathways in combination with an HDI. We propose that the combination of an HDI with a dual inhibitor of the MAPK and PI3K pathways may be an effective way to treat Ras-mutant cancers.

## RESULTS

### Short-term romidepsin treatment in combination with inhibitors of the MAPK and PI3K pathways induces apoptosis preferentially in cancer cell lines harboring mutant Ras

We previously reported that activation of the MAPK pathway is a resistance mechanism to the HDI romidepsin, via phosphorylation and degradation of the proapoptotic protein Bim [11]. Since mutant Ras proteins activate both the MAPK and PI3K pathways, and as both pathways have been shown to prevent apoptosis, we decided to investigate whether combining romidepsin with inhibitors of both pathways would induce cell death in Ras mutant cell lines. To more closely approximate the dosing schedule of romidepsin given to patients, we treated cells for 6 h with romidepsin alone or in combination with the inhibitors, then removed the medium and incubated the cells in romidepsin-free medium in the absence or presence of the inhibitors for an additional 42 h, after which cells were stained with annexin/PI and assayed by flow cytometry [16]. We previously observed that this schedule of romidepsin exposure gives a broader range of efficacy in cell lines, with the profile of susceptible lines more closely resembling the clinical profile [16].

**Figure 1:**
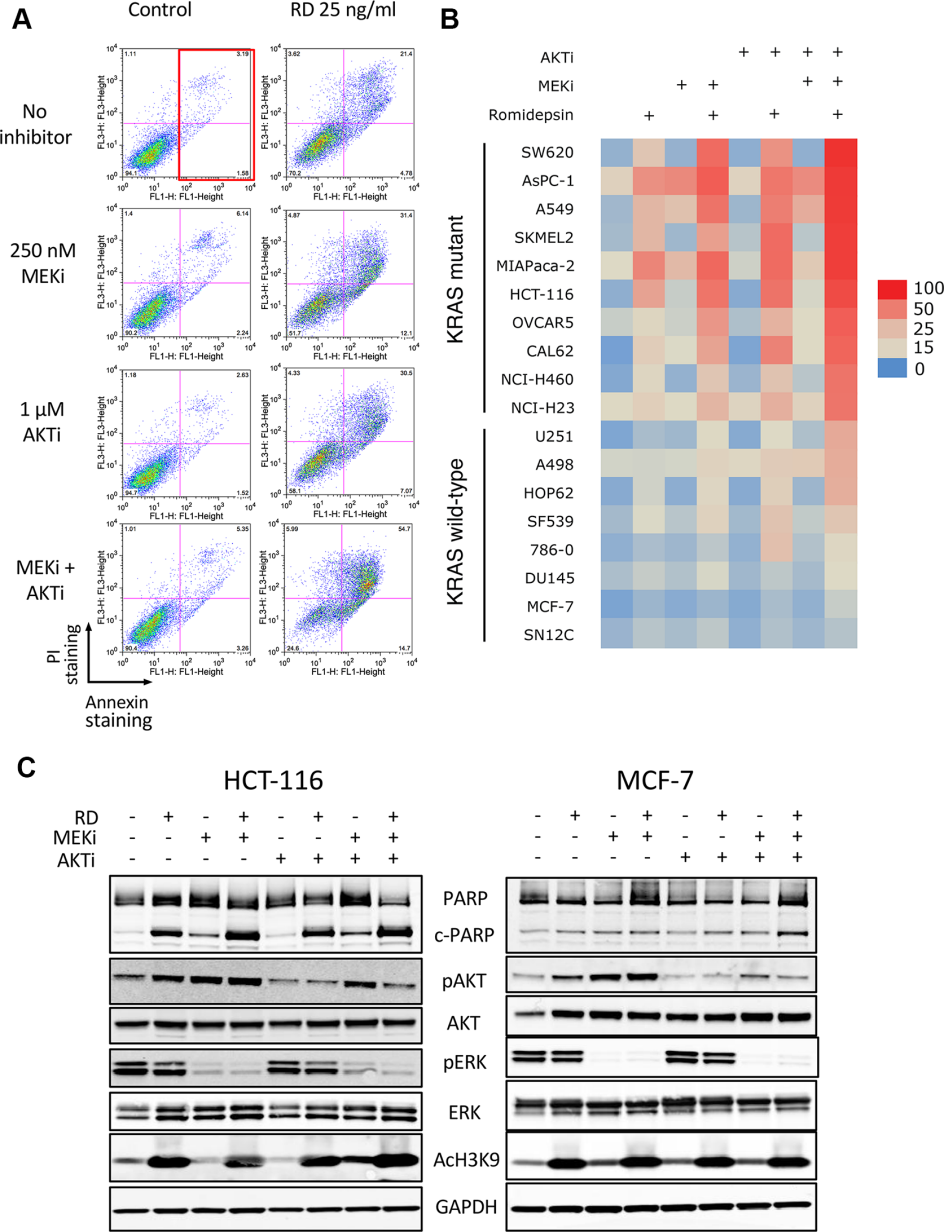
Romidepsin in combination with a MEK and an AKT inhibitor is selectively toxic to cells harboring mutant Ras (**A**) HCT-116 cells were treated for 6 h with 25 ng/ml romidepsin (RD) alone or in combination with 250 nM of the MEK inhibitor PD-0325901 (MEKi) and/or 1 μM of the AKT inhibitor MK-2206 (AKTi). The medium was subsequently removed and cells were incubated in romidepsin-free medium in the absence or presence of the inhibitors for an additional 42 h, after which cells were stained with annexin/PI and assayed by flow cytometry. The red box denotes annexin-positive cells. (**B**) Heat map constructed using the percentage of annexin-positive cells determined for each treatment in Ras mutant and Ras wild-type cells. Data from at least 3 separate experiments was compiled. (**C**) Ras mutant HCT-116 cells and Ras wild-type MCF-7 cells were exposed for 6 h to 25 ng/ml romidepsin (RD) alone or in combination with 250 nM of MEKi and/or 1 μM of the AKTi. The medium was subsequently removed and cells were incubated in romidepsin-free medium in the absence or presence of the inhibitors for an additional 18 h, after which the cells were harvested. Cell lysates were prepared and separated via SDS-PAGE and transferred to nitrocellulose membranes. The membranes were subsequently probed with antibodies to PARP and cleaved PARP (c-PARP), phorphorylated AKT (Ser473) (pAKT), total AKT, phospho-ERK, (Thr202, Tyr204) (pERK), total ERK (ERK) and acetylated histone H3 (Lysine 9) (AcH3K9). GAPDH served as a loading control. At least 2 independent experiments were performed.

For our initial screening assay, we used 250 nM of the MEK inhibitor (MEKi) PD-0325901 and 1 μM of the AKT inhibitor (AKTi) MK-2206 alone or in combination with 25 ng/ml romidepsin, screening a series of 18 cell lines harboring mutant or wild-type Ras (Ras mutation status is provided in Table 1). Results with the HCT-116 cell line are shown in Figure 1A. The red box indicates the region where cells were counted as annexin positive. In the absence of romidepsin, the MEK and/or AKT inhibitors for 48 h alone resulted in little to no increased cell death compared to control cells. When romidepsin was combined with the inhibitors, we observed a marked increase in cell death (second and third plots on the right), from 13% with romidepsin alone to 52 and 27%, when combined with MEKi or AKTi, respectively. However, when both inhibitors were combined with romidepsin, annexin staining was detected in 74% of cells (lower right plot). Annexin data from all 14 cell lines were used to generate the heat map shown in Figure 1B. As can be observed, cell lines harboring mutant Ras were more sensitive to combinations with romidepsin compared to cell lines bearing wild-type Ras. The average percentage of annexin positive cells was 69.1% in the Ras mutant lines compared to 21.1% in the wild-type Ras lines when cells were treated with the triple combination (*P <* 0.0001). The sensitivity to the combination of romidpesin, MEKi and AKTi was observed regardless of the Ras mutation (see Table 1), despite the fact that specific KRAS mutations have been shown to variably signal through the MAPK and PI3K pathways [17].

**Table 1:** Cell line origin and Ras mutation

Cell line	Origin	Ras mutation (AA change)
SW620	Colon	KRAS G12V
A549	Lung	KRAS G12S
SKMEL2	Skin	NRAS Q61R
HCT-116	Colon	KRAS G13D
CAL62	Thyroid	KRAS G12R
OVCAR5	Ovary	KRAS G12V
NCI-H460	Lung	KRAS Q61H
HCI-H23	Lung	KRAS G12C
AsPC-1	Pancreas	KRAS G12D
MIAPaCa-2	Pancreas	KRAS G12C
U251	Brain	Wild-type
A498	Kidney	Wild-type
HOP92	Lung	Wild-type
SF539	Brain	Wild-type
786-0	Kidney	Wild-type
MCF-7	Breast	Wild-type
SN12C	Kidney	Wild-type
DU145	Prostate	Wild-type

The activity of the pathway inhibitors in both Ras mutant and wild-type lines was subsequently verified by examining expression of downstream effectors of the MAPK and PI3K pathway in the KRAS mutant line HCT-116 and the Ras wild-type line MCF-7. As shown in Figure 1C, MEKi treatment abrogated ERK phosphorylation, AKTi treatment abrogated AKT phosphorylation and romidepsin treatment increased histone H3 acetylation of lysine 9 in both cell lines. As reported by other groups, we observed an increase in AKT phosphorylation after treatment with the MEKi [18, 19]. Interestingly, we found that short-term romidepsin treatment similarly increased phosphorylation of AKT. In agreement with the results obtained from the annexin assay, increased PARP cleavage was found in HCT-116 cells treated with romidepsin or romidepsin combinations, while little PARP cleavage was observed in the MCF-7 cell line, which was largely resistant to all treatments.

**Figure 2:**
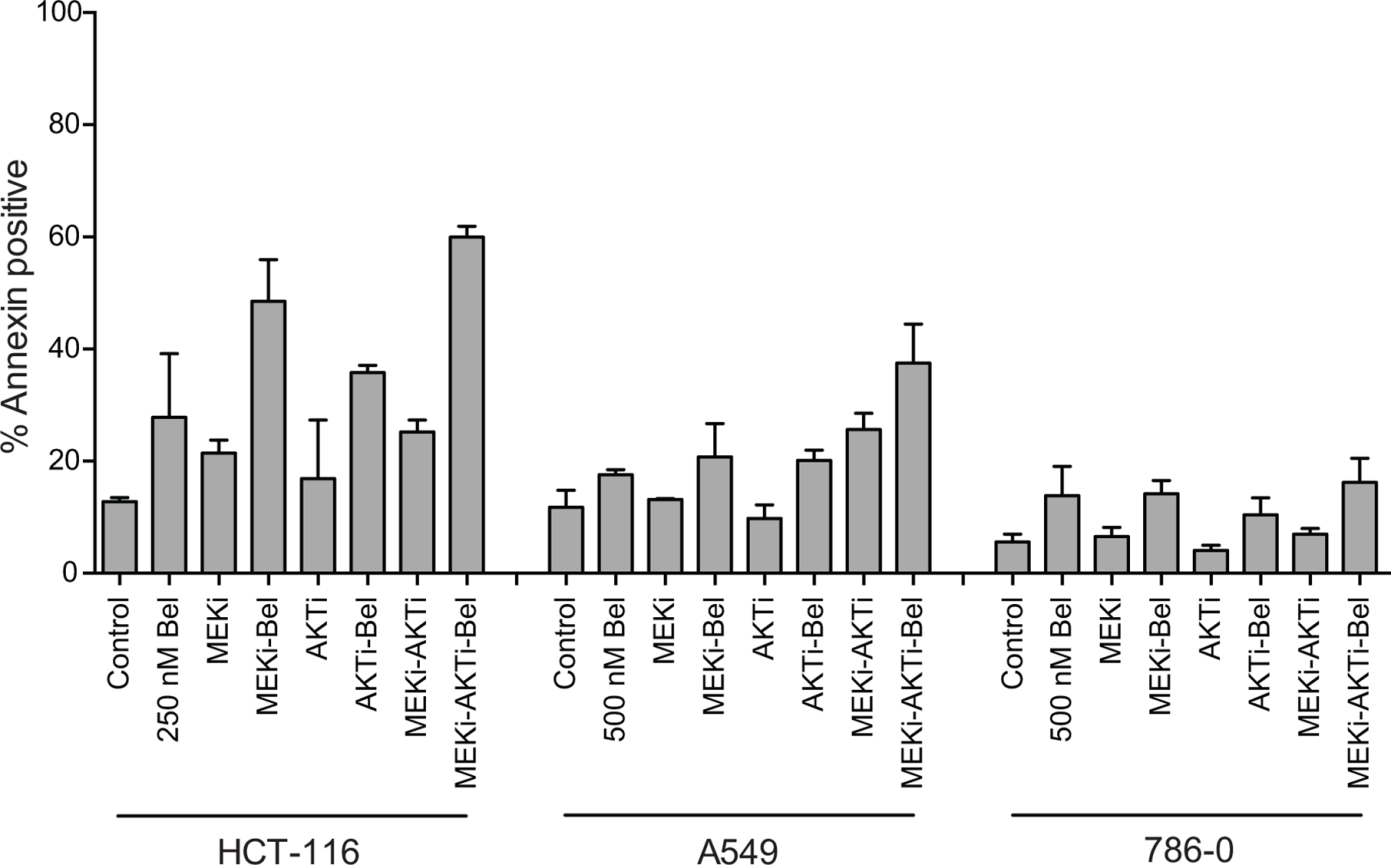
Belinostat in combination with a MEK inhibitor and an AKT inhibitor is effective in Ras mutant cancers Ras mutant HCT-116 and A549 cells and Ras wild-type 786-0 cells were treated with 250 or 500 nM belinostat (Bel) for 48 h alone or in combination with 250 nM of the MEK inhibitor PD-0325901 (MEKi) and/or 1 μM of the AKT inhibitor MK-2206 (AKTi) after which cells were stained with annexin/PI and assayed by flow cytometry. Bars represent mean percent annexin-positive cells with error bars representing standard deviation from the mean. At least 3 separate experiments were performed.

To verify that the observed apoptosis was not specific to romidepsin, we also examined the effects of combining a 48 h treatment of the HDI belinostat with the MEKi and/or the AKTi in a subset of the cell lines. We chose belinostat, as a recent clinical trial reported administration of belinostat as a 48 h continuous infusion, with serum levels of 1 μM consistently achieved [20]. Similar to results achieved with romidepsin after a 6 h exposure, a 48 h treatment of belinostat at a concentration of 250 to 500 nM was relatively non-toxic to HCT-116, A549 or 786-0 cells. However, when combined with inhibitors of the MAPK and/or PI3K pathway for the duration of the 48 h treatment, increased apoptosis was observed in the Ras mutant cell lines, while little effect was observed in the wild-type Ras line, 786-0 (Figure 2). Compared to the results with romidepsin in HCT-116 cells found in Figure 1A and 1B, the belinostat combinations do appear to be less effective than those with romidepsin.

Thus, combination of a clinically-relevant, short-term romidepsin treatment with inhibitors of the MAPK and PI3K pathways was shown to induce apoptosis selectively in cells that harbor a Ras mutation.

### Apoptosis induced by romidepsin combinations is caspase-mediated and requires bak and bax

**Figure 3:**
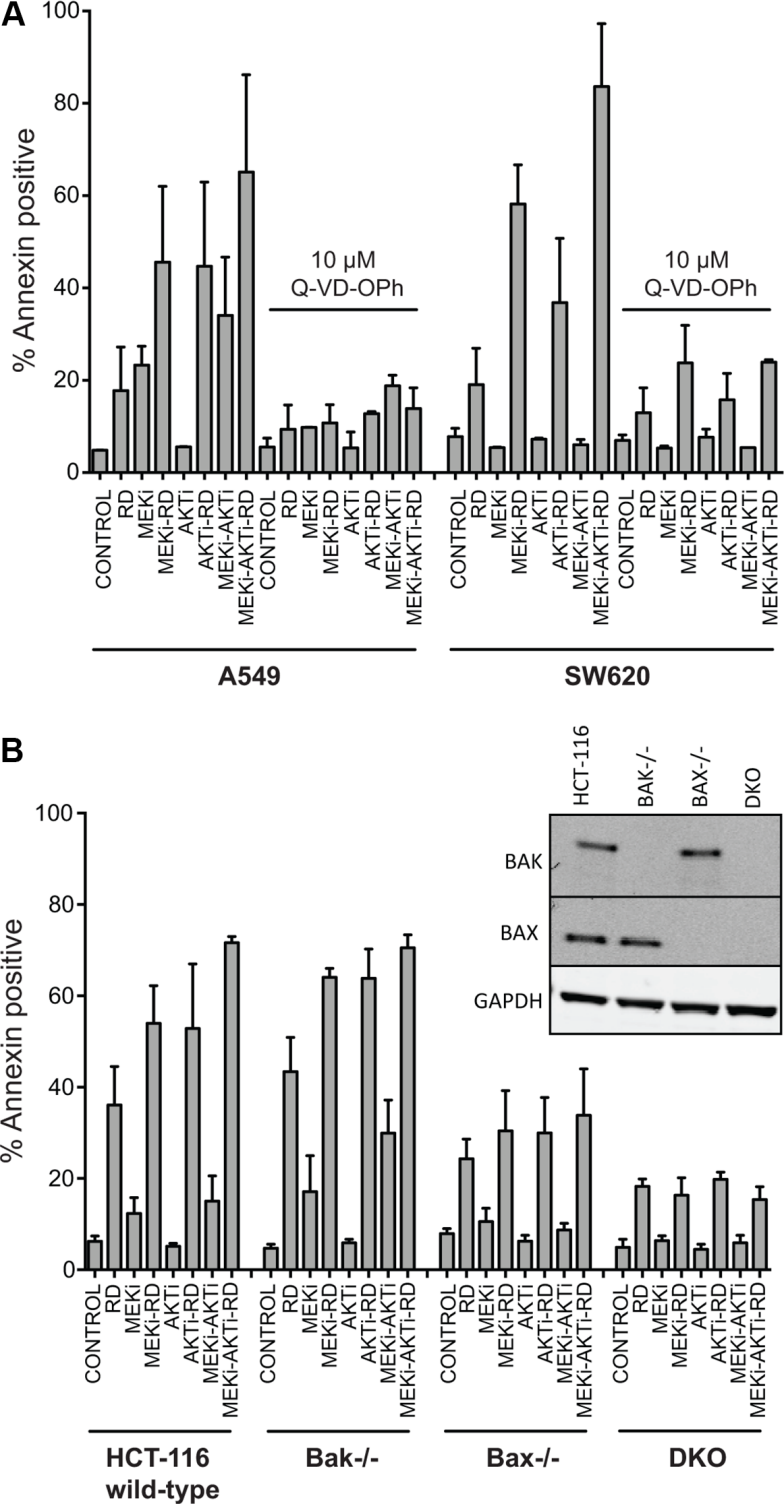
Apoptosis induced by treatment with romidepsin in combination with a MEK and an AKT inhibitor in KRAS mutant cells is caspase-dependent and requires Bak and Bax (**A**) A549 and SW620 cells were treated for 6 h with 25 ng/ml romidepsin (RD) alone or in combination with 250 nM of the MEKi and/or 1 μM of the AKTi in the presence or absence of 10 μM of the pan-caspase inhibitor Q-VD-OPh. The medium was subsequently removed and cells were incubated in romidepsin-free medium in the absence or presence of the inhibitors for an additional 42 h, after which cells were stained with annexin/PI and assayed by flow cytometry. Bars represent mean percent annexin-positive cells with error bars representing standard deviation from the mean. At least 3 separate experiments were performed. (**B**) Parental HCT-116 cells or cells lacking Bak (Bak−/−), Bax (Bax−/−) or both proteins (double knock-out, DKO) were subjected to treatment with romidepsin (RD) for 6 h alone or in combination with 250 nM MEKi and/or 1 μM of the AKTi. The medium was subsequently removed and cells were incubated in romidepsin-free medium in the absence or presence of the inhibitors for an additional 42 h, after which cells were stained with annexin/PI and assayed by flow cytometry. Bars represent mean percent annexin-positive cells with error bars representing standard deviation from the mean. At least 3 separate experiments were performed. 3B Inset: Bak and Bax expression was confirmed in the cell lines with GAPDH serving as a loading control.

Caspase inhibitors have previously been shown to prevent apoptosis mediated by HDIs [21, 22] and we examined their role in the romidepsin combinations. Annexin staining was measured in the romidepsin combinations on KRAS mutant SW620 and A549 cell lines in the absence or presence of the pan-caspase inhibitor Q-VD-OPh. As shown in Figure 3A, apoptosis was nearly completely abrogated when cells were incubated with the romidepsin combinations in the presence of 10 μM Q-VD-OPh, confirming the role of caspases. Since studies have shown that the Bcl-2 family of proteins is important for HDI-mediated apoptosis [11, 22–24], we examined the effects of the romidepsin combinations in control HCT-116 cells or HCT-116 cells lacking Bak, Bax or both proteins (double knock-out, DKO). As seen in Figure 3B, we found that loss of Bak and Bax results in near complete abolition of apoptosis, suggesting that the observed cell death occurs via the mitochondrial pathway. The previously reported presence or absence of Bak and Bax expression in the parental line and in the knock-out lines, respectively, was confirmed (Figure 3B inset) [25].

### Treatment with romidepsin and MAPK and PI3K inhibitors induces changes in the expression of pro- and antiapoptotic proteins

**Figure 4:**
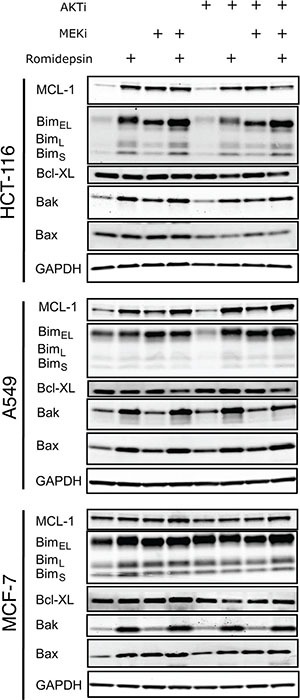
Short-term romidepsin treatment in combination with a MAPK and a PI3K inhibitor induces changes in expression of pro- and antiapoptotic proteins (A) Ras mutant HCT-116 and A549 cells and Ras wild-type MCF-7 cells were exposed to romidepsin (RD) alone or in combination with 250 nM of MEKi and/or 1 μM of the AKTi. The medium was subsequently removed and cells were incubated in romidepsin-free medium in the absence or presence of the inhibitors for an additional 18 h, after which cells were harvested. Cell lysates were prepared and separated via SDS-PAGE and transferred to nitrocellulose membranes that were subsequently probed with antibodies to MCL-1, Bim, Bak, Bax and Bcl-XL. GAPDH served as a loading control. At least 2 independent experiments were performed.

Since our data implicated the mitochondrial pathway of apoptosis, we subsequently examined the effect of the combination treatments on the expression of MCL-1, Bim, Bcl-XL, Bak and Bax—all members of the Bcl-2 family of proteins. Treatment with romidepsin as well as the MEKi induced higher expression of the proapoptotic protein Bim, of which there are 3 isoforms: Bim_EL_, Bim_L_ and Bim_S_ (Figure 4). Direct phosphorylation of Bim_EL_ by ERK is known to negate its proapoptotic function and prime the protein for degradation by the proteasome [5, 7]. We and others found that phosphorylation of Bim is associated with romidepsin resistance [11, 26]. Romidepsin alone or in combination with the inhibitors slightly induced Bak and Bax expression (Figure 4). Interestingly, we also noticed that MCL-1 expression was induced by romidepsin alone in the Ras mutant lines as well as in combination with the inhibitors. The MEKi also induced MCL-1 expression, but the AKTi did not. Other reports have also shown the induction of MCL-1 expression by a MEKi in A549 cells [27]. In MCF-7 cells, which have wild-type Ras, none of the treatments seemed to have a significant effect on MCL-1 expression. The combination of romidepsin with both of the inhibitors resulted in a slight decrease in levels of Bcl-XL in the Ras mutant lines. The ability of proapoptotic proteins to overcome the expression of MCL-1 induced by romidepsin in Ras mutant cells may ultimately dictate cell fate.

### A dual inhibitor of the MAPK and PI3K pathways induces apoptosis in Ras mutant cancers when combined with romidepsin

**Figure 5:**
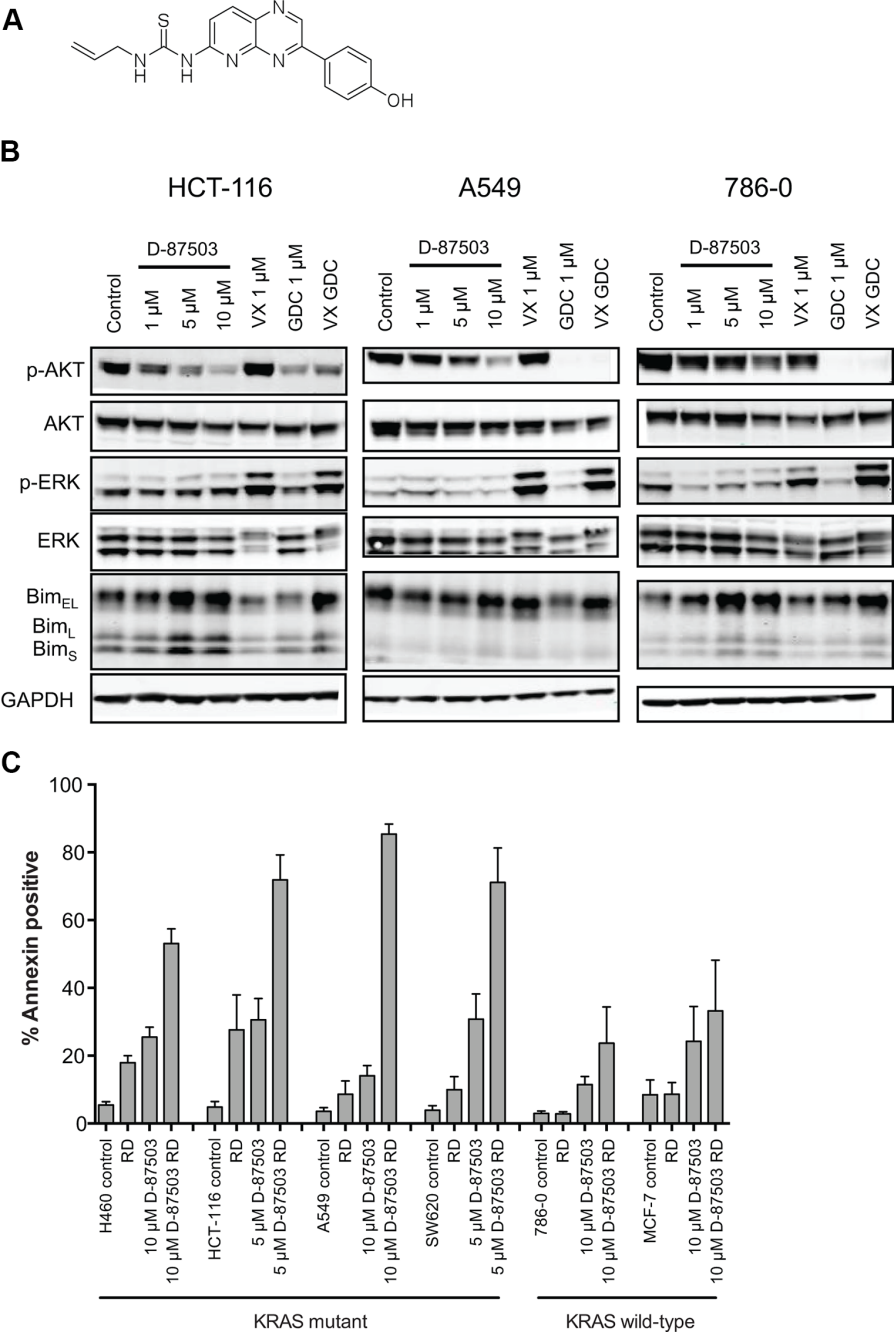
A dual inhibitor of the MAPK and PI3K pathways induces apoptosis in KRAS mutant cell lines when combined with romidepsin (**A**) Chemical structure of D-87503. (**B**) Ras-mutant HCT-116 and A549 cells and Ras wild-type 786-0 cells were treated with 1, 5 or 10 μM D-87503, 1 μM Vx-11e (VX), 1 μM GDC-0941 (GDC) or both Vx-11e and GDC-0941 (VX GDC) for 24 h. Cell lysates were subsequently prepared, separated via SDS-PAGE and transferred to nitrocellulose membranes that were probed with antibodies to phosphorylated AKT (Ser473) (pAKT), total AKT, phospho-ERK, (Thr202, Tyr204) (pERK), total ERK (ERK) and Bim. GAPDH served as a loading control. At least 2 independent experiments were performed. (**C**) Ras-mutant NCI-H460, HCT-116, A549 and SW620 cells as well as Ras wild-type 786-0 and MCF-7 cells were treated for 6 h with 25 ng/ml romidepsin (RD) alone or in combination with 5 or 10 μM D-87503. The medium was subsequently removed and cells were incubated in romidepsin-free medium in the absence or presence of D-87503 for an additional 42 h, after which cells were stained with annexin/PI and assayed by flow cytometry. Bars represent mean of annexin-positive cells with error bars representing standard deviation from the mean. At least 3 separate experiments were performed.

Several studies have shown that inhibition of both the MAPK and PI3K pathways is more effective in Ras mutant cancers than either inhibitor alone [28, 29], leading to the development of dual inhibitors of the two pathways [30, 31]. We thus synthesized D-87503, a dual inhibitor of both ERK and PI3K [31]; the structure is shown in Figure 5A. The ability of a 24 h treatment of D-87503 to inhibit the PI3K and MAPK pathways was compared to the specific ERK inhibitor Vx-11e and the PI3K inhibitor GDC-0941 individually and in combination in Figure 5B. Inhibition of the MAPK pathway and the PI3K pathway was confirmed by reappearance of dephosphorylated Bim and dephosphorylation of AKT, respectively. We also noted an in increase in pERK expression after Vx-11e treatment, a finding consistent with that of a previous report [32]. D-87503 inhibited both pathways at approximately 5–10 μM in HCT-116, A549, and 786-0 cells, with a decrease observed in pAKT along with an increase in Bim expression. Combination studies were also performed with romidepsin. We examined induction of apoptosis in a subset of mutant and wild-type Ras cell lines treated with romidepsin in combination with 5 or 10 μM D-87503. As shown in Figure 5C, while D-87503 was minimally toxic alone, combination with romidepsin resulted in a marked increase in apoptosis in H460, HCT-116, A549, and SW620 cells expressing mutant Ras. Less apoptosis was observed when wild-type Ras 786-0 and MCF-7 cells were treated with the combination. This suggests that the combination of a dual inhibitor of the MAPK and PI3K pathways with a histone deacetylase inhibitor such as romidepsin may offer a unique approach to targeting cancers with a RAS mutation.

## DISCUSSION

HDIs have entered the anticancer armamentarium, with 4 agents approved by the U.S. Food and Drug Administration, three for T-cell lymphomas. As epigenetic agents, the HDIs offer a unique mechanism of action that is as yet not fully understood, but whose efficacy we have sought to translate to solid tumors. In the course of these studies, we have found that HDIs can overcome the anti-apoptotic machinery of cells bearing Ras mutations when used in conjunction with inhibitors of pathways activated by mutant Ras and that the intrinsic apoptotic pathway is of critical importance in promoting the observed cell death.

Treatment for cancers harboring Ras mutations remains a major unmet need in cancer – mutation of Ras is one of the most common mutations found in sequencing studies of the cancer genome. Reported in over 90% of pancreatic cancers, 33% of colon cancers, 32% of lung cancers, and 31% of biliary tract cancers, KRAS is mutated in some of the most treatment-resistant solid tumors [33]. In pancreatic cancer, KRAS is considered the earliest oncogenic event [34]. Ras is a protein previously considered undruggable because it has no ligand-binding pocket to target, as found in many other kinase inhibitors that have been successfully targeted. However, the NCI has launched a major new initiative, bringing in many new investigators to develop therapies to target RAS, and some of the earlier concepts may be changing [2].

We have here identified a new approach to targeting KRAS-mutated cancers using the histone deacetylase inhibitor romidepsin. Romidepsin is the most potent HDI developed to date, with *in vitro* efficacy in the nanomolar range, even with short drug exposures. While the mechanism of HDI efficacy in cancer is not fully understood, effects including induction of genes that promote cell death, DNA damage, reactive oxygen species release, and acetylation of cytoplasmic proteins have been suggested [35]. HDI-mediated changes in the expression of Bcl-2 family proteins have been shown to be very important indicators of whether cell death results from HDI exposure [11, 22, 24, 26, 36, 37]. Since the antiapoptotic protein MCL-1 was induced by romidepsin in our study, this could represent a resistance mechanism to short-term romidepsin exposure in solid tumors. In order to ultimately induce apoptosis, a sufficient pro-apoptotic signal may be needed to overcome this mechanism. The fact that the combination of the MEK and AKT inhibitors appeared to blunt the induction of MCL-1 by romidepsin treatment could contribute to the efficacy of this combination. In support of this hypothesis is the fact that other groups have also shown that antiapoptotic proteins are a potential target in KRAS-mutant cancers [38, 39]. We are currently exploring the contribution of individual pro- and antiapoptotic proteins to the efficacy of romidepsin and other HDIs.

Although Ras is known to signal through multiple pathways, the PI3K and MAPK pathways are the most studied and compounds targeting these pathways are in clinical development [2]. However, these compounds are generally not sufficiently toxic in Ras mutant cancers and clinical trials have been disappointing [40, 41]. To increase their efficacy, some groups have proposed combinations with HDIs. Ablation of AKT activity by inhibitors or AKT1 knockdown sensitized a series of colorectal cancers to the class I HDAC inhibitor 4SC-2 [42]. Jokinen and Koivunen demonstrated increased PARP cleavage in HCT-116 cells when entinostat was combined with either the MEK inhibitor CI-1040 or the PI3K inhibitor ZSTK474 [43]. It is interesting to note that entinostat and 4SC-202 both inhibit the primarily nuclear Class I HDACs and that romidepsin is also primarily a Class I inhibitor by virtue of its potency against those isoforms. However, they found significant cell death with the combination of the MEK and PI3K inhibitors even without the addition of entinostat. Ischenko et al. examined the combination of a MEK inhibitor (GSK1120212) and a PI3K inhibitor (BEZ235) with the HDI trichostatin A in pancreatic cancer [44]. They noted that treating pancreatic cancer cell lines with either a MEK inhibitor or a PI3K inhibitor had a significant cytostatic effect, but, when the two inhibitors were combined with trichostatin A, significant apoptosis was observed [44]. When they further examined a set of lung cancer cell lines with mutant or wild-type KRAS, treatment with the MEK/PI3K/HDAC inhibitor combination elicited apoptosis only in the KRAS mutant cell lines. We extend these earlier studies, demonstrating apoptosis in Ras mutant cell lines when an HDI is combined with a dual inhibitor of the MAPK and PI3K pathways. Although an explanation for the sensitivity of Ras mutant lines to the combination remains to be determined, ongoing studies are evaluating the hypothesis that HDIs impair the glutamine utilization pathway, which has been shown to be important for KRAS-mutant cancers.

In conclusion, we find that Ras mutant cancers can be sensitized to a clinically-relevant treatment of romidepsin *in vitro* by combining romidepsin with inhibitors of the MAPK and PI3K pathways. A dual inhibitor of these pathways was also found to have robust activity when combined with romidepsin. Although relatively few such dual inhibitors are currently reported—current options lack significant potency—our results combining these inhibitors with romidepsin suggest a unique efficacy in Ras mutant cancers that to date have few therapeutic options. We continue to investigate combination therapies for Ras mutant cancers that include HDIs as key components.

## MATERIALS AND METHODS

### Cell culture

Ras mutant (SW620, HCT-116, SKMEL2, OVCAR5, A549, NCI-H460, NCI-H23) and Ras wild-type (U251, SF539, SN12C, A498, 786-0, HOP92, MCF-7) cell lines were obtained from the National Cancer Institute Tumor Drug Screen (Bethesda, MD). AsPC-1 and MIA PaCa-2 pancreatic cancer cell lines were purchased from ATCC (Masassas, VA); CAL62 cells were from the Leibniz Institute DSMZ-German Collection of Microorganisms and Cell Cultures (Braunschweig, Germany). Cell line origin and Ras mutation site are provided in Table 1. HCT-116 cells lacking Bax were a gift from Dr. Bert Vogelstein [45], while HCT-116 cells lacking Bak or both Bak and Bax were a gift from Dr. Richard Youle [25]. Cell lines were authenticated by STR-DNA technology (Idexx Bioresearch, Columbia, MO) and fingerprints were as previously reported. All cell lines were kept in RPMI-1640 (Mediatech, Manassas, VA) supplemented with 10% FBS, glutamine and antibiotics and maintained in 5% CO_2_ at 37°C.

### Chemicals

Romidepsin was obtained from the Developmental Therapeutics Program of the National Cancer Institute (Bethesda MD). The MEK inhibitor PD-0325901, the AKT inhibitor MK-2206, the ERK inhibitor Vx-11e and the PI3K inhibitor GDC-0941 were purchased from ChemieTek (Indianapolis, IN). Q-VD-OPh was from R&D Systems (Minneapolis, MN). The dual pathway inhibitor, D-87503, was synthesized in-house by the Chemical Biology Laboratory, Center for Cancer Research (Frederick, MD).

### Flow cytometry

After indicated treatment, cells were harvested and incubated with fluorescein isothiocyanate (FITC)-labeled human recombinant Annexin V (eBioscience, San Diego, CA) and propidium iodide (PI) according to the manufacturer's instructions. Samples were read on a FACSort Flow Cytometer (Becton Dickinson, San Jose, CA) and percent annexin positive cells were calculated using FlowJo software.

### Immunoblot analysis

Treated cells were harvested and sonicated for at least 45 s, after which samples were centrifuged to remove nuclei and unlysed cells. Protein was loaded into 4–12% Bis-Tris NuPAGE gels, subjected to electrophoresis, and transferred to 0.2 μm pore size nitrocellulose membranes. Blots were probed with antibodies to one or more of the following proteins: total PARP (#9542), cleaved PARP (#5625), total AKT (#9272), phospho-AKT Ser473 (#9271), total ERK (#9101), phospho-ERK Thr202, Tyr204 (#9102), Bim (#2933), Bak (#6947), Bax (#5023), MCL-1 (#4572), and Bcl-XL (#2762) (all from Cell Signaling Technology, Danvers, MA). Acetylated histone H3 (Lysine 9) antibody (#06-942) was from EMD Millipore (Billerica, MA). Glyceraldehyde 3-phosphate dehydrogenase (GAPDH) antibody (#05-50118) was purchased from American Research Products (Waltham. MA). Proteins were visualized with the Odyssey System (LI-COR, Lincoln, NE) using a 1:10000 dilution of IRDye secondary antibodies (LI-COR).

### Statistical analysis

Where noted, a student's *T-test* was performed with differences considered significant where *P <* 0.05.
